# Supplementary material for: Mg2+ is a Missing Link in Plant Cell Ca2+ Signalling and Homeostasis—A Study on Vicia faba Guard Cells
Source: Int J Mol Sci. 2020 May 27;21(11):3771. doi: 10.3390/ijms21113771 (PMC7312177; doi:10.3390/ijms21113771)
Supplement: Supplementary file 1 [file ijms-21-03771-s001.pdf]

Supplementary Figure 1

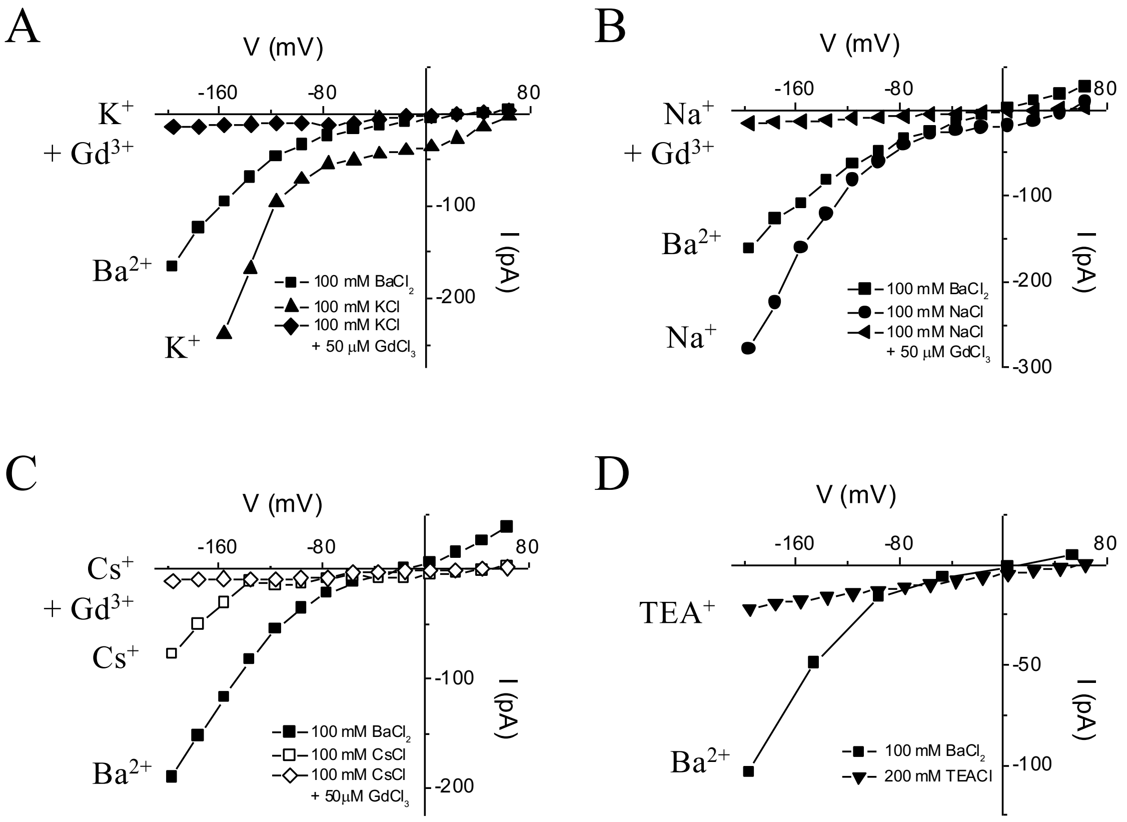

Suppl. Figure 1: In the absence of MgATP, Guard cell's HACCs are permeable to monovalent cations such as K^+, Na^+, Cs^+ but not TEA^+. All experiments were conducted in the whole cell configuration where *V. faba* GCPs were held at -56 mV. (**A**) Superimposed I-V plots in the presence of 100 mM $BaCl_2$ (■), 100 mM KCl (▲) or 100 KCl + 0.05 mM $GdCl_3$. (**B**) Superimposed I-V plots in the presence of 100 mM $BaCl_2$ (■), 100 mM NaCl (●) or 100 NaCl + 0.05 mM $GdCl_3$. (**C**) Superimposed I-V plots in the presence of 100 mM $BaCl_2$ (■) or 100 mM CsCl (□) or 100 CsCl + 0.05 mM $GdCl_3$ (◇). (**D**) Superimposed I-V plots in the presence of 100 mM $BaCl_2$ (■) or 100 TEACl (▼).

Supplementary Figure 2

A

```
PLANT_371-506     TTFFSKFCYCLWWGLQNLSTLGQGLQTSTYPGEVLFSIAIAVAGLLLFALLIGNMQTYLQ
5u6o_341_471      -SWGKQYSYALFKAMSHMLCIGYGAQAPVSMSDLWITMLSMIVGATCYAMFVGHATALIQ
                   :: .::.*.*:..:.::  :* * *:..  .:: :::   :.*   :*:::*:  : :*

PLANT_371-506     SLTVRLEEMRIKRRDSEQWMHHRSLPQNLRERVRRYDQYKWLETRGVDEENIVQSLPKDL
5u6o_341_471      SLDSSRRQYQEKYKQVEQYMSFHKLPADMRQKIHDYYEHRY-QGKIFDEENILNELNDPL
                  **     : . * .: **:* ...** ::*:.:. * ::.: : . .*****::.* . *

PLANT_371-506     RRDIKRHLCLNLVRRV
5u6o_341_471      REEIVNFNCRKLVATM
                  * :* .. * :**  :
```

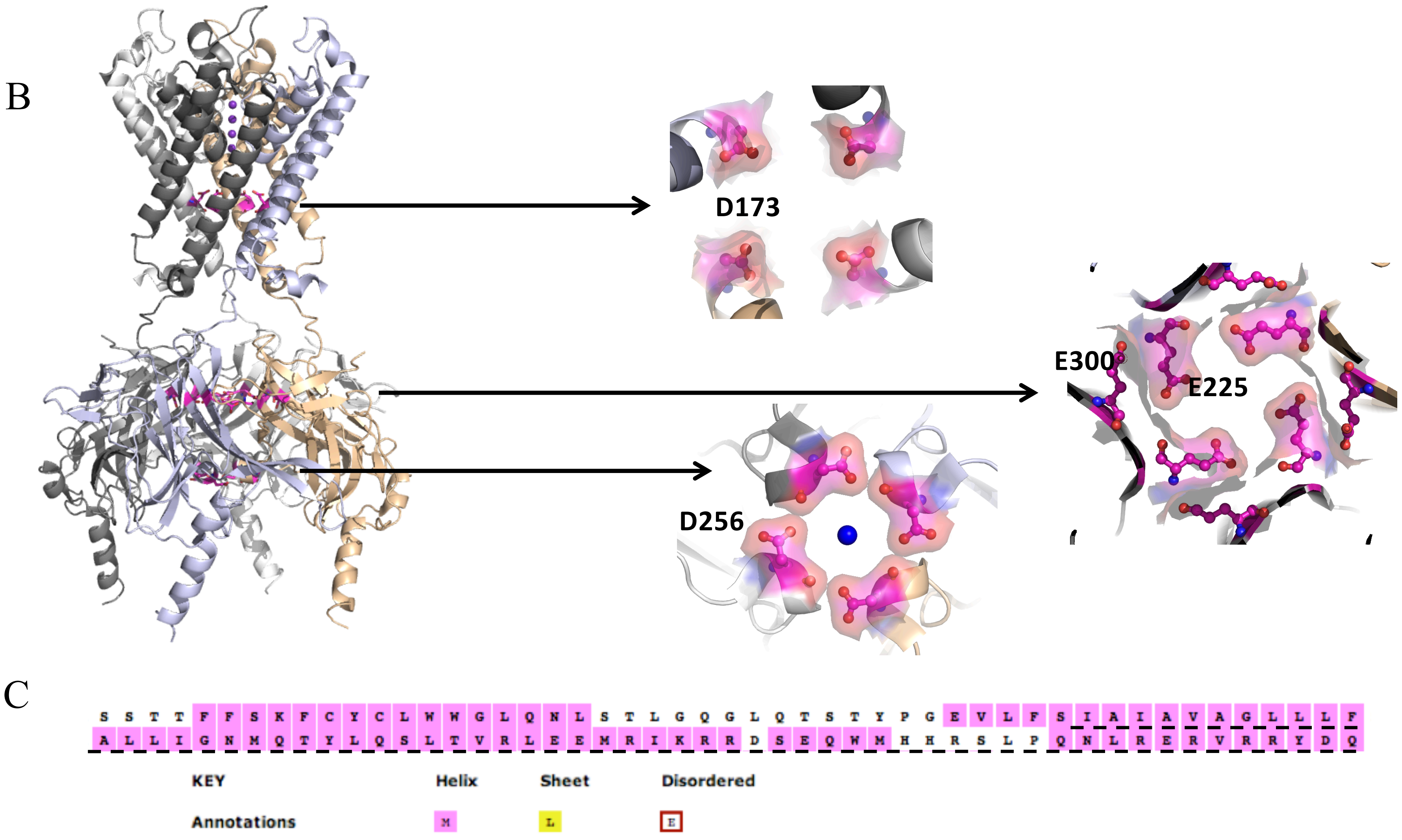

Suppl. Figure 2:

(A) Alignment of the *Arabidopsis thaliana* CNGC8 pore region and the Kir2.2 (PDB accession number 5u6o_341_471).

(B) Mechanism of inward rectifying by magnesium ions. Tao *et al.* (Science 2009, 326, 1668-1674) have shown that inward rectifying through Mg^{2+} can be explained by the ion binding to negatively charged regions in the pore (formed by D173) and in the cytoplasmic regulatory domains (D256 and E300/E225). The crystal structure of the inward rectifying potassium channel Kir2.2 (Tao, 2009; PDB entry 3JYC) is shown in ribbon presentation. The four subunits are colour-coded. Potassium ions in the channel are shown as magenta spheres. Negatively charged residues that bind the Mg^{2+} - mimic Sr^{2+} in the crystal structure are shown in pink in their molecular surface.

(C) Sequences of pore-forming transmembrane helix (TM) from AtCNGCs. The TM is marked with a dashed line.

Models were built using Swiss-Model (Waterhouse *et al*. (Nucleic Acids Res. 2018, 46, W296-W303)).
